# Tissue Factor‐Targeted ImmunoPET Imaging and Radioimmunotherapy of Anaplastic Thyroid Cancer

**DOI:** 10.1002/advs.201903595

**Published:** 2020-05-17

**Authors:** Weijun Wei, Qiufang Liu, Dawei Jiang, Haitao Zhao, Christopher J. Kutyreff, Jonathan W. Engle, Jianjun Liu, Weibo Cai

**Affiliations:** ^1^ Department of Nuclear Medicine Institute of Clinical Nuclear Medicine Renji Hospital, School of Medicine Shanghai Jiao Tong University State Key Laboratory of Oncogenes and Related Genes Shanghai Cancer Institute 1630 Dongfang Rd Shanghai 200127 China; ^2^ Departments of Radiology and Medical Physics University of Wisconsin–Madison Madison WI 53705 USA; ^3^ Department of Nuclear Medicine Fudan University Shanghai Cancer Center Fudan University 270 Dongan Rd Shanghai 200032 China; ^4^ University of Wisconsin Carbone Cancer Center Madison WI 53705 USA

**Keywords:** anaplastic thyroid cancer, immunoPET, radioimmunotherapy, theranostics, tissue factor

## Abstract

Anaplastic thyroid cancer (ATC) is the most aggressive subtype of thyroid cancers with a dismal prognosis. It is aimed to explore a new biomarker and devise a marker‐dependent theranostic pair for ATC. Flow cytometry is used to determine tissue factor (TF) expression in thyroid cancer cell lines. ALT‐836, a TF‐specific monoclonal antibody, is radiolabeled with ^64^Cu to develop ^64^Cu‐NOTA‐ALT‐836. The diagnostic utility is assessed by immuno‐positron emission tomography (immunoPET) imaging in ATC models. To facilitate total surgical removal of orthotopic ATCs, a near‐infrared fluorescent imaging probe IRDye 800CW‐ALT‐836 is designed. As the therapeutic component, ^131^I‐ALT‐836 is further developed and the radioimmunotherapy (RIT) efficacy of this agent is interrogated in orthotopic ATC models. The results demonstrate that TF is highly expressed on the ATC cell line THJ‐16T. ^64^Cu‐NOTA‐ALT‐836 immunoPET imaging clearly delineates both subcutaneous and orthotopic ATCs, with a peak tumor uptake of 19.93 ± 2.17% ID per g (*n* = 3) and 37.20 ± 1.71% ID per g (*n* = 3), respectively. Fluorescent imaging with IRDye 800CW‐ALT‐836 facilitates the total resection of orthotopic ATCs. Moreover, ^131^I‐ALT‐836 RIT prolongs the survival of ATC‐bearing mice. Taken together, TF is a promising marker for ATC and successive use of ^64^Cu‐NOTA‐ALT‐836 and ^131^I‐ALT‐836 can realize precise management of ATC.

## Graphical abstract

Anaplastic thyroid cancer (ATC) is a lethal disease. This study reports tissue factor (TF) as a potential biomarker for ATC and develops a TF‐targeted toolbox consisting of ^64^Cu‐NOTA‐ALT‐836 and ^131^I‐ALT‐836. While ^64^Cu‐NOTA‐ALT‐836 immunoPET readily detects and stratifies ATCs, ^131^I‐ALT‐836 radioimmunotherapy efficiently inhibits the growth of the tumors.

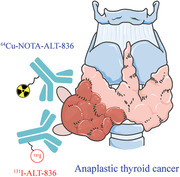

## 1 Introduction

Anaplastic thyroid cancer (ATC) is a rare but deadly disease with a dismal prognosis. The median overall survival (OS) of ATC patients receiving multimodal therapy and palliative treatment was 21 months and 3.9 months, respectively. Despite the therapeutic effect of multimodal therapy, the median OS of patients with ATC was far from satisfactory.^[^ ^1^ ^]^ Recent progress in understanding the molecular pathogenesis of thyroid cancers has led to the clinical translation of molecularly targeted therapies.^[^ ^2^, ^3^, ^4^ ^]^ However, adverse effects and drug resistance associated with these regimens limit their broad clinical applications. Immunotherapy using immune checkpoint inhibitors showed therapeutic effect in sparse cases,^[^ ^5^, ^6^, ^7^, ^8^ ^]^ so the net benefit needs to be carefully characterized.^[^ ^9^ ^]^ Unlike well‐differentiated thyroid cancers, ATCs are virtually refractory to radioiodine therapy due to loss of sodium iodide symporter (NIS). Along with the above progress, continuous attempts have been made to re‐differentiate ATC to enhance NIS uptake,^[^ ^10^ ^]^ but the clinical effect of these agents remains to be determined.

In recent years, antibody therapeutics are increasingly being approved for cancer treatment.^[^ ^11^, ^12^ ^]^ Although therapeutic antibodies are among the mainstream of cancer therapy, practical challenges lie in how to efficiently stratify patients before drug administration and how to precisely monitor the therapeutic responses of antibody drugs. Immuno‐positron emission tomography (immunoPET), which integrates the specificity of the antibody vector and the sensitivity of the PET technique, emerges as a paradigm‐shifting molecular imaging modality.^[^ ^13^ ^]^ Various targets have been exploited to develop immunoPET probes, including receptor tyrosine kinases,^[^ ^14^ ^]^ and T cell markers.^[^ ^15^ ^]^ Besides its role in detecting heterogeneous tumors, immunoPET can select candidates who may benefit from antibody‐based therapies, such as radioimmunotherapy (RIT). RIT is developed by conjugating therapeutic radioisotopes with tumor‐targeting antibodies and allows delivery of a high dose of therapeutic radioactivity to the tumor cells.^[^ ^16^, ^17^ ^]^ Traditionally, RIT was used for radiosensitive tumors, such as lymphomas and leukemia. Two CD20‐targeting RIT agents, ^131^I‐tositumomab (Bexxar), and ^90^Y‐ibritumomab tiuxetan (Zevalin), are among the FDA‐approved drugs for treating B cell lymphomas and have yielded considerable efficacy.^[^ ^18^, ^19^ ^]^ For leukemia, several RIT agents targeting CD33, CD45, or CD66 have been developed.^[^ ^16^ ^]^ RIT agents labeled with *α*‐emitters also hold great promise.^[^ ^20^ ^]^ More recently, RIT has been extended to treat solid tumors such as neuroblastoma,^[^ ^21^, ^22^, ^23^ ^]^ and colorectal cancer.^[^ ^24^ ^]^

Bearing the unsatisfactory management status of ATC in mind, we turned to seek biomarkers that can be manipulated to develop theranostic probes for ATCs.^[^ ^25^, ^26^ ^]^ Tissue factor (TF) plays a pivotal role in mediating hemostasis and inflammatory diseases.^[^ ^27^ ^]^ Recent evidence has elucidated that TF serves as an alternative target for cancer therapy.^[^ ^28^ ^]^ Several antibody therapeutics targeting TF have been proposed,^[^ ^29^, ^30^ ^]^ including a chimeric monoclonal antibody (mAb) ALT‐836.^[^ ^31^ ^]^ We reported that molecular imaging probes derived from ALT‐836 have shown great potential in diagnosing pancreatic cancer and breast cancer.^[^ ^32^, ^33^, ^34^ ^]^ More recently, we demonstrated the feasibility of pancreatic cancer theranostics using ^86/90^Y‐labeled ALT‐836.^[^ ^35^ ^]^ In the current study, we first elucidate whether TF serves as a tumor marker for advanced thyroid cancers and then investigate the diagnostic utility of TF‐specific ^64^Cu‐NOTA‐ALT‐836 in subcutaneous and orthotopic ATC models. We further develop a fluorescent probe IRDye 800CW‐ALT‐836 and investigate the feasibility of image‐guided surgery with this probe. Finally, ^131^I‐ALT‐836 is conjugated and the therapeutic efficacy of ^131^I‐ALT‐836 RIT will be explored in orthotopic ATC models.

## 2 Results

### 2.1 Tissue Factor Is a Biomarker for Advanced Thyroid Cancers

**Figure 1:**
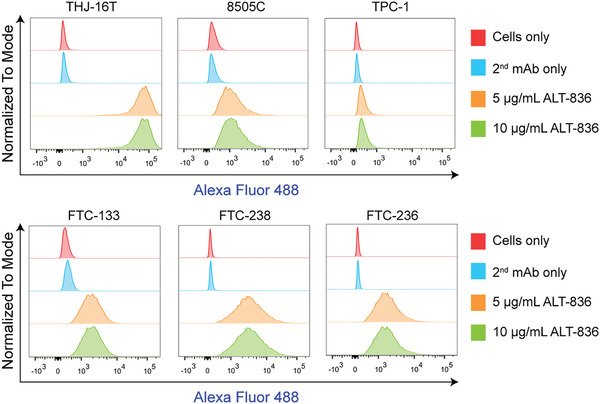
Detection of tissue factor levels in thyroid cancer cell lines by flow cytometry. This panel included two anaplastic thyroid cancer cell lines (THJ‐16T and 8505C), one papillary thyroid cancer cell line (TPC‐1), and three follicular thyroid cancer cell lines (FTC‐133, FTC‐238, and FTC‐236).

We first screened six thyroid cancer cell lines by flow cytometry. The results showed five of the six included cell lines were TF‐positive, including two ATC cell lines (THJ‐16T and 8505C) and three follicular thyroid cancer cell lines (FTC‐133, FTC‐236, and FTC‐238). TPC‐1, a papillary thyroid cancer cell line, was TF‐negative (**Figure** **1**). Considering the gloomy course of ATCs and the strongest TF expression on the outer membrane of THJ‐16T cells, this cell line was used to establish ATC models for subsequent imaging and therapy studies.

### 2.2 ImmunoPET Imaging of Subcutaneous ATCs

**Figure 2:**
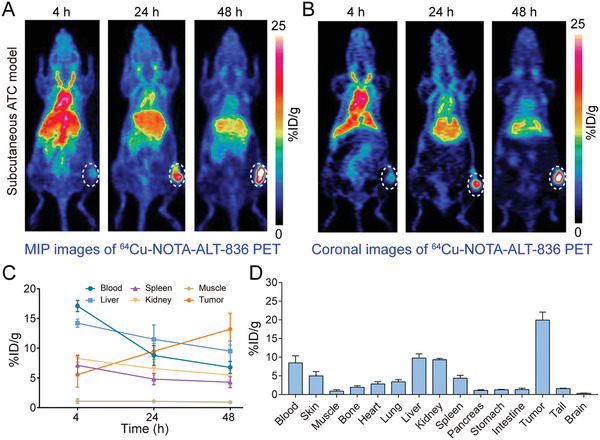
^64^Cu‐NOTA‐ALT‐836 immunoPET imaging in subcutaneous anaplastic thyroid cancer (ATC) models. A) Representative maximum intensity projection (MIP) and B) coronal images showed the overall and focal distribution of ^64^Cu‐NOTA‐ALT‐836 across the body at different time‐points. Tumors were indicated by white dashed circles. C) Time–activity curves showed the dynamic change of ^64^Cu‐NOTA‐ALT‐836 in the blood pool and in the major organs/tissues. D) Biodistribution data.

^64^Cu‐NOTA‐ALT‐836 was developed with high decay‐corrected radiochemical yield (>80%) and excellent radiochemical purity (>99%). ImmunoPET imaging in subcutaneous ATC models showed a prominent accumulation of ^64^Cu‐NOTA‐ALT‐836 in tumors than in mesenchymal tissues. Both maximum intensity projection (MIP, **Figure** **2**A) and coronal (Figure 2B) immunoPET images showed the clear delineation of THJ‐16T tumors at 48 h post‐injection of the radiotracer. Quantitative analysis of the PET data revealed the dynamic change of the radiotracer in the blood circulation and in other selected organs (Figure 2C). Specifically, tumor uptake of ^64^Cu‐NOTA‐ALT‐836 increased across the imaging period with the highest uptake of 13.20 ± 2.67% ID per g (*n* = 4) achieved at 48 h after radiotracer administration. In comparison, uptake in other organs was low except that in the liver (9.53 ± 1.69% ID per g, *n* = 4). The in vivo imaging results were corroborated by the ex vivo biodistribution data (Figure 2D), which revealed a comparable liver uptake (9.77 ± 1.12% ID per g, *n* = 3) but a higher tumor uptake (19.93 ± 2.17% ID per g, *n* = 3).

**Figure 3:**
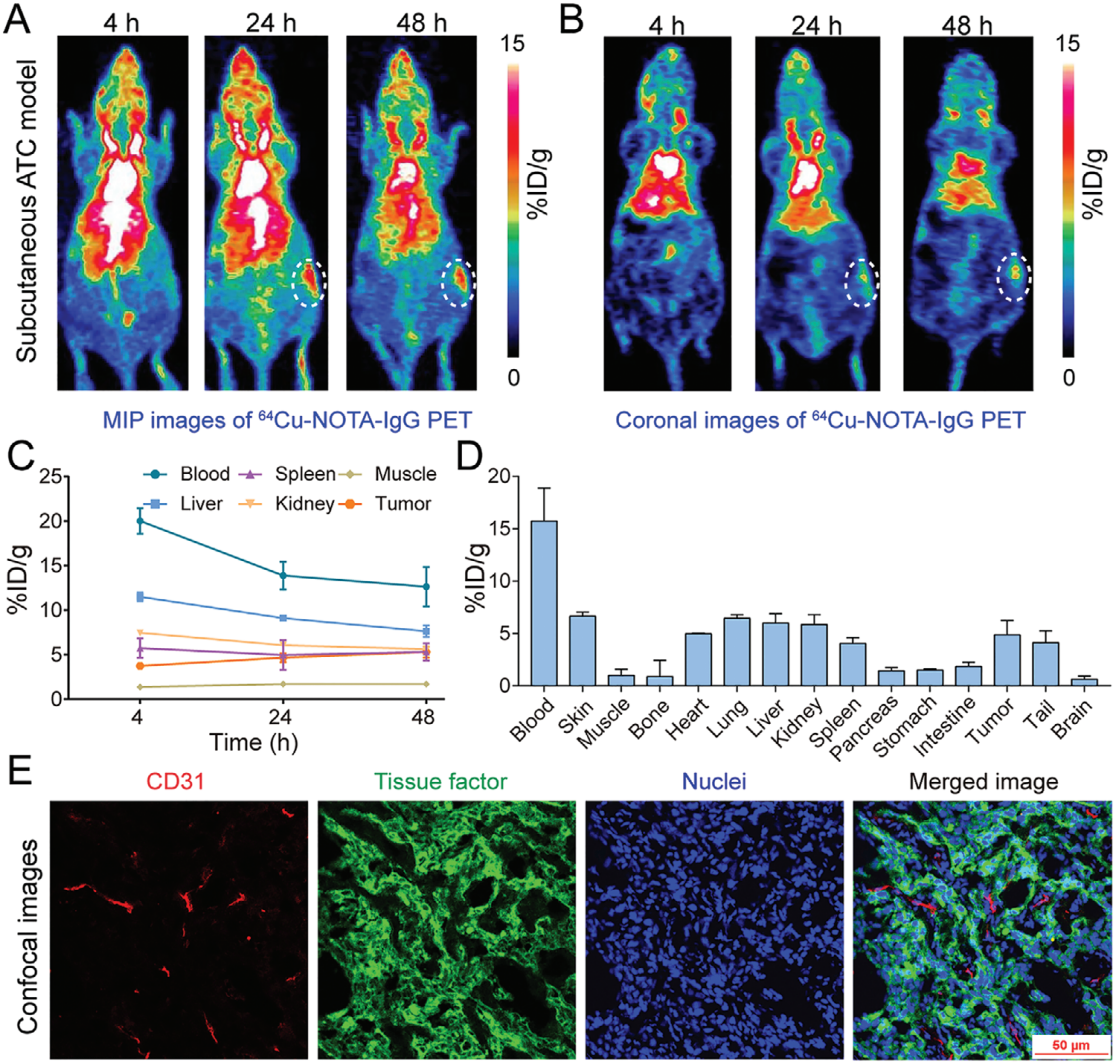
^64^Cu‐NOTA‐IgG immunoPET imaging in subcutaneous anaplastic thyroid cancer (ATC) models. A) Representative maximum intensity projection (MIP) and B) coronal images showed the overall and focal distribution of ^64^Cu‐NOTA‐IgG at different time‐points. Tumors were indicated by white dashed circles. C) Time–activity curves showed the dynamic change of ^64^Cu‐NOTA‐IgG in the blood pool and in the major organs/tissues. D) Biodistribution data. E) Immunofluorescent staining and imaging of the collected tumor tissue. Blood vessels were stained with CD31 (red), tissue factor was stained with ALT‐836 (green), and nuclei were stained with DAPI (blue).

We then asked if immunoPET imaging with a ^64^Cu‐labeled nonspecific human IgG (i.e., ^64^Cu‐NOTA‐IgG) was able to delineate subcutaneous THJ‐16T tumors. As shown in **Figure** **3**, although MIP and coronal images showed visible uptake of ^64^Cu‐NOTA‐IgG in the tumor area, the uptake was comparable to or lower than the liver uptake. Quantitatively, tumor uptake of ^64^Cu‐NOTA‐IgG plateaued at 48 h with a value of 5.30 ± 0.62% ID per g (*n* = 3), which was significantly lower than 13.20 ± 2.67% ID per g (*n* = 4) achieved by ^64^Cu‐NOTA‐ALT‐836 (*p* = 0.0044). From the region of interest (ROI) and biodistribution data (Figure 3C,D), it is clear that the majority of ^64^Cu‐NOTA‐IgG resided in the circulation at 48 h post‐injection. Laser confocal immunofluorescence scanning of the stained tumor tissue showed that a large proportion of the THJ‐16T cells were TF‐positive (Figure 3E). These results indicate the potency of ^64^Cu‐NOTA‐ALT‐836, but not ^64^Cu‐NOTA‐IgG, in noninvasively diagnosing subcutaneous ATCs.

### 2.3 ^64^Cu‐NOTA‐ALT‐836 ImmunoPET Imaging of Orthotopic ATCs

**Figure 4:**
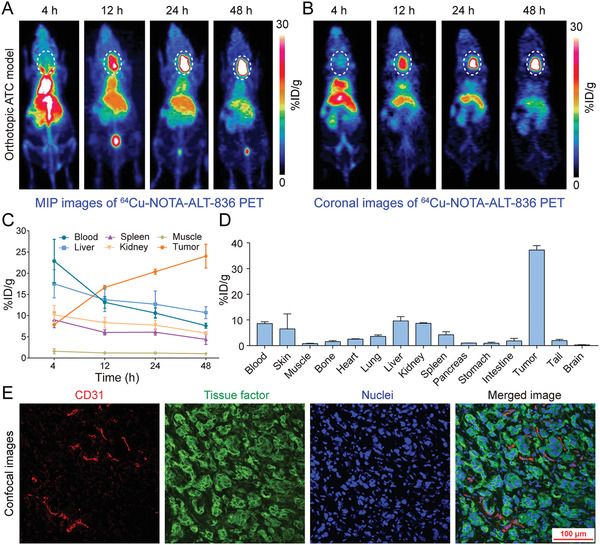
^64^Cu‐NOTA‐ALT‐836 immunoPET imaging in orthotopic anaplastic thyroid cancer (ATC) models. A) Representative maximum intensity projection (MIP) and B) coronal images showed the overall and focal distribution of ^64^Cu‐NOTA‐ALT‐836 at different time‐points. Tumors were indicated by white dashed circles. C) Time–activity curves showed the dynamic change of ^64^Cu‐NOTA‐ALT‐836 in the blood pool and in the major organs/tissues. D) Biodistribution data. E) Immunofluorescent staining and imaging of the collected tumor tissue. Blood vessels were stained with CD31 (red), tissue factor was stained with ALT‐836 (green), and nuclei were stained with DAPI (blue).

The above results further prompted us to investigate the potency of ^64^Cu‐NOTA‐ALT‐836 in diagnosing orthotopic ATCs. Tumor burden was monitored by fluorescent imaging with IRDye 800CW‐pertuzumab, a near‐infrared (NIR) probe we previously described.^[^ ^26^ ^]^ Two weeks after tumor inoculation, serial fluorescent imaging demonstrated focal signals in the neck areas, indicating rapid formation and growth of the orthotopic tumors (Figure S1, Supporting Information). ^64^Cu‐NOTA‐ALT‐836 immunoPET imaging of these mice another two weeks later showed dramatic uptake of the tracer in the thyroid areas (**Figure** **4**A,B). Quantitatively, tumor accumulation of ^64^Cu‐NOTA‐ALT‐836 increased in a time‐dependent manner, with the uptake value at 4, 12, 24, and 48 h was 7.87 ± 0.31, 16.67 ± 0.46, 20.37 ± 0.61, and 24.03 ± 2.80% ID per g, respectively (*n* = 3). In comparison, uptake in the blood pool, liver, spleen, and kidney decreased over the imaging course and the organ with the highest uptake at 48 h was the liver (10.70 ± 1.42% ID per g, *n* = 3; Figure 4C). It is notable that tumor accumulation of ^64^Cu‐NOTA‐ALT‐836 was significantly higher in orthotopic ATC models than in subcutaneous ATC models (24.03 ± 2.80% ID per g [*n* = 3] vs 13.20 ± 2.67% ID per g [*n* = 4], *p* = 0.0035). The tumor‐targeting ability of ^64^Cu‐NOTA‐ALT‐836 was further confirmed by the biodistribution study, which revealed an average tumor uptake of 37.20 ± 1.71% ID per g (*n* = 3) with the uptake in other organs less than 10% ID per g (Figure 4D). Immunofluorescence staining and imaging of the collected tumor tissue showed luxuriant supplying vessels and abundant membrane expression of TF on the THJ‐16T cells (Figure 4E). The above evidence demonstrates the efficacy of ^64^Cu‐NOTA‐ALT‐836 immunoPET in diagnosing orthotopic ATCs.

### 2.4 Image‐Guided Surgery with IRDye 800 CW‐ALT‐836

**Figure 5:**
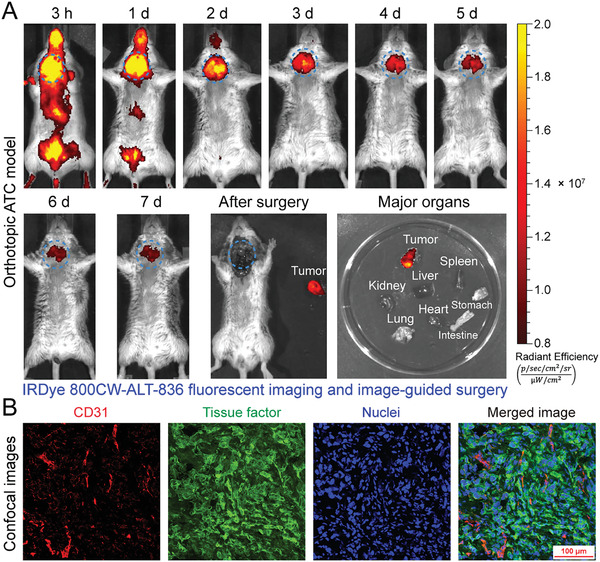
Image‐guided surgery of orthotopic anaplastic thyroid cancers (ATCs) using IRDye 800CW‐ALT‐836. A) Serial fluorescent imaging after injection of IRDye 800CW‐ALT‐836, followed by image‐guided resection of the orthotopic tumor seven days later. Ex vivo fluorescent imaging showed an intense fluorescent signal in the tumor but not in other collected organs. The tumor was indicated by blue dashed circles. B) Immunofluorescent staining and imaging of the orthotopic tumor. Blood vessels were stained with CD31 (red), tissue factor was stained with ALT‐836 (green), and nuclei were stained with DAPI (blue).

While ^64^Cu‐NOTA‐ALT‐836 immunoPET may comprehensively delineate TF expression across the body, it is not suitable for bedside image‐guided surgery. To facilitate total thyroidectomy, we further developed a NIR probe, IRDye 800CW‐ALT‐836, and investigated whether IRDye 800CW‐ALT‐836 NIR imaging can guide intraoperative surgery. In R2G2 mice bearing orthotopic ATCs, NIR imaging with the developed fluorescent probe readily visualized the orthotopic tumors and the tumor‐to‐background ratio increased as the time went on (**Figure** **5**A; Figure S2, Supporting Information). Seven days later, the tumor was resected in an image‐guided manner and the surgical margins were negative. Post‐mortem fluorescence imaging of the tumor together with other resected organs showed an exclusive fluorescence signal within the tumor but no detectable signal in other organs (Figure 5A), indicating the superior targeting ability of IRDye 800 CW‐ALT‐836 towards ATCs and the feasibility of image‐guided surgery of the orthotopic ATCs. Immunofluorescence staining and imaging of the orthotopic tumor from a representative R2G2 mouse also showed a strong expression of TF on the surface of tumor cells (Figure 5B).

### 2.5 ^131^I‐ALT‐836 Radioimmunotherapy of ATCs

**Figure 6:**
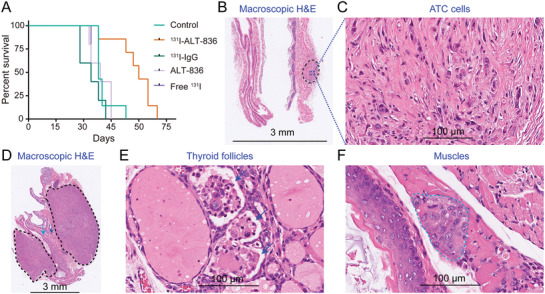
Radioimmunotherapy (RIT) of orthotopic anaplastic thyroid cancers (ATCs). A) Kaplan–Meier survival curves. A significant difference in median survival was observed between mice treated with ^131^I‐ALT‐836 and normal saline (control group) (*p* = 0.0089), or ^131^I‐IgG (*p* = 0.0016). When compared to the control group, no significant survival improvement was observed for mice receiving ALT‐836 (*p* = 0.7773) or free ^131^I treatment (*p* = 0.8056). B) Macroscopic H&E staining of an orthotopic tumor from the ^131^I‐ALT‐836 treatment group. The tumor (black dashed circle) did not compress the adjacent trachea. C) The tumor tissue is devoid of any thyroid follicles. D–F) Macroscopic H&E staining of archived tumor tissue in the control group showed irregular bilateral tumors (black dashed circles), effaced and infiltrated thyroid follicles (blue arrows), and invaded muscle (dark green arrow and dashed circle).

Motivated by the prominent tumor uptake of ^64^Cu‐NOTA‐ALT‐836 and IRDye 800 CW‐ALT‐836, we further developed ^131^I‐ALT‐836 and explored the effect of ^131^I‐ALT‐836 RIT in orthotopic ATC models. ^131^I‐labeling of ALT‐836 resulted in an overall labeling yield of 64.44 ± 0.08% (*n* = 3). As detected by instant thin‐layer chromatography (iTLC), the radiochemical purity of ^131^I‐ALT‐836 was 99.06 ± 0.01% (*n* = 3, Figure S3A, Supporting Information). Protein integrity according to the high‐performance liquid chromatography (HPLC) test was 100% with a retention time of 13 min for the main peak (i.e., ^131^I‐ALT‐836) (Figure S3B–D, Supporting Information). Bioluminescence imaging before initiation of treatment showed comparable tumor burden in the control group and ^131^I‐ALT‐836 treatment group (Figure S4A,B, Supporting Information). Treatment with ^131^I‐ALT‐836 was safe because no weight loss was observed. Since it was unable to measure the volume of the orthotopic tumors longitudinally, we performed fluorescent imaging with IRDye 800CW‐ALT‐836 26 days after ^131^I‐ALT‐836 injection to monitor the tumor burden. When compared to the control group, ^131^I‐ALT‐836 treatment significantly reduced the tumor uptake of IRDye 800CW‐ALT‐836 (Figure S4C,D, Supporting Information), indicating a single administration of ^131^I‐ALT‐836 substantially saturated and/or downregulated TF expression in the tumors. Repeated IRDye 800CW‐ALT‐836 fluorescent imaging 41 days after the therapy further consolidated the above observation (Figure S4E,F, Supporting Information). RIT with a single injection of ^131^I‐ALT‐836 significantly extended the survival of the tumor‐bearing mice, whereas no other treatment options including ALT‐836 monotherapy showed any therapeutic benefit (**Figure** **6**A). More specifically, a significant difference in the median survival was observed between mice treated with ^131^I‐ALT‐836 and saline (60 days vs 38 days, *p* = 0.0089), ^131^I‐IgG (60 days vs 34 days, *p* = 0.0016), ALT‐836 (60 days vs 39 days, *p* = 0.0054), or free ^131^I (60 days vs 39 days, *p* = 0.0055).

Mice receiving ^131^I‐ALT‐836 treatment showed either no tumor or just small tumors at necropsy. Hematoxylin and eosin (H&E) staining showed no morphologic features of follicular cells but obvious fibrosis inside the small tumor of the ^131^I‐ALT‐836 treatment group (Figure 6B,C). In comparison, H&E staining of the tumor of the control group showed bilateral tumors effacing thyroid follicles and infiltrating adjacent muscles (Figure 6D–F). H&E staining from other groups also showed similar aggressive patterns of orthotopic ATCs (Figure S5, Supporting Information). ^131^I‐ALT‐836 RIT caused degradation and necrosis of a small proportion of liver cells (Figure S6, Supporting Information), but this novel treatment did not cause any obvious damage to other major organs (Figure S7, Supporting Information). We also stained the normal mouse tissues and found that expression of TF is quite low in organs such as heart, liver, kidney, and muscle (Figure S8, Supporting Information). There was positive staining in the lung and spleen, which was largely localized to the blood cells. Finally, we performed biodistribution studies to compare the overall distribution and tumor‐targeting capacity of ^131^I‐ALT‐836 and ^131^I‐IgG (Figure S9, Supporting Information). The results showed a significantly higher tumor uptake of ^131^I‐ALT‐836 than that of ^131^I‐IgG (38.79 ± 18.08 vs 25.54 ± 10.22, *n* = 4 for each group). Although the uptake of ^131^I‐IgG in the parenchymal organs (e.g., heart, lung, and spleen) was higher than the corresponding uptake of ^131^I‐ALT‐836, there was no statistical difference in the uptake value in these organs. Both ^131^I‐ALT‐836 and ^131^I‐IgG were stable within two days since there was no abnormal accumulation of free ^131^I in the stomach.

These results together demonstrated that regardless of the differentiation status, ^131^I‐ALT‐836 could specifically target THJ‐16T tumors and ^131^I‐ALT‐836 RIT could induce tumor regression and prolong the survival of the tumor‐bearing mice.

## 3 Discussion

In developing antibody‐based theranostic approaches for human malignancies, two crucial factors are the binding affinity of the antibody moiety and the antigen expression level. Most of the antibody‐derived probes explored in clinical practice are targeting cluster of differentiation antigens,^[^ ^17^, ^36^ ^]^ receptor tyrosine kinases,^[^ ^14^ ^]^ and enzymes.^[^ ^37^ ^]^ Recent advances have shown that TF is a promising diagnostic marker for several categories of malignancies, including pancreatic cancer,^[^ ^32^, ^34^ ^]^ and breast cancer.^[^ ^33^ ^]^ In this work, we first reported the abundant expression of TF in advanced thyroid cancer cell lines, indicating the potential of TF as a biomarker for advanced thyroid cancers. We then developed and characterized the value of ^64^Cu‐NOTA‐ALT‐836 and ^131^I‐ALT‐836 in diagnosing and treating ATCs in preclinical mouse models.

One merit of ^64^Cu‐NOTA‐ALT‐836 immunoPET is its ability to detect advanced thyroid cancers irrespective of NIS expression. TF‐positive cases as selected by ^64^Cu‐NOTA‐ALT‐836 immunoPET will presumably benefit from TF‐targeted therapies, including antibody and antibody‐derived therapies. In our case, ^131^I‐ALT‐836 showed preliminary therapeutic effects in orthotopic ATC models. The preclinical evidence warrants further clinical investigation of ^64^Cu‐NOTA‐ALT‐836 in the first place. If clinical ^64^Cu‐NOTA‐ALT‐836 immunoPET could diagnose advanced thyroid cancers and the immunoPET signal correlates well with TF expression revealed by immunohistochemistry, then it is rational to investigate the therapeutic value of ^131^I‐ALT‐836 in the selected cases. Currently, combinatorial treatment incorporating surgery (when feasible) and chemoradiotherapy is the standard of care for ATC patients.^[^ ^1^, ^38^ ^]^ Complete surgical resection of the tumors has been shown to improve the survival of ATC patients.^[^ ^39^ ^]^ Our preclinical results also showed that NIR imaging with IRDye 800CW‐ALT‐838 is a robust method to facilitate the total resection of the orthotopic tumors. However, assessing the impact of the dye‐to‐mAb ratio on the circulation profile of the developed fluorescent probe is very necessary before clinical translation.^[^ ^40^ ^]^ In several clinical trials, a dye‐to‐mAb molar ratio of 2.0–2.3:1 was used in developing the fluorescent probes.^[^ ^41^, ^42^, ^43^ ^]^

Most of the ongoing clinical RIT trials are using ^131^I and ^90^Y (an almost exclusively *β*‐particle‐emitting radionuclide). We also used ^131^I in this preclinical study. However, ^131^I‐labeled mAbs are usually catabolized and disconnected after internalization,^[^ ^44^, ^45^ ^]^ as a result, ^131^I‐tyrosine and free ^131^I will be released into the blood circulation which may subject family members and the public under unnecessary radiation exposure. Future studies may develop radiometal‐labeled ALT‐836 which may retain inside the tumor cells after antigen‐antibody complex internalization. Of the various radiometals, ^90^Y and ^177^Lu are of choice.^[^ ^18^, ^35^, ^46^ ^]^ For micrometastases or diseases significantly involving bone marrow, *α*‐particle‐emitting radionuclides, such as ^211^At (*t*_1/2_ = 7.2 h) and ^225^Ac (*t*_1/2_ = 10 d), are attractive choices for their high linear energy transfer within a few diameters (50–90 µm) and high potency in inducing DNA double‐strand breaks.^[^ ^47^, ^48^, ^49^ ^]^ Additionally, pretargeted RIT may be harnessed to relieve the side effects and maximize the therapeutic index.^[^ ^50^ ^]^

## 4 Conclusion

TF is a promising biomarker for ATCs. TF‐targeted theranostics using ^64^Cu‐NOTA‐ALT‐836 and ^131^I‐ALT‐836 could optimize the diagnosis and treatment of ATCs in preclinical settings.

## 5 Experimental Section

##### Cell Lines and Flow Cytometry

Six thyroid cancer cell lines (THJ‐16T, 8505C, TPC‐1, FTC‐133, FTC‐236, and FTC‐238) used in this study were kindly provided by Dr. Heather Hardin (Department of Pathology and Laboratory Medicine, University of Wisconsin–Madison).^[^ ^26^ ^]^ To better monitor the tumor burden, the THJ‐16T^Luc^ cell line was constructed using a pGMLV‐CMV‐Lu lentivirus luciferase reporter (Genomeditech). All the cell lines were cultured following the recommended protocols. To detect cell surface expression of TF by flow cytometry, 1 × 10^6^ cells for each sample were collected and washed with cold phosphate‐buffered saline (PBS, HyClone). The cells were then resuspended in flow cytometry staining buffer (Invitrogen) and incubated with primary antibodies (5 or 10 µg mL^−1^ of ALT‐836) on ice for 45 min, followed by washing with PBS and incubation with Alexa Fluor 488‐labeled goat anti‐human IgG (5 µg mL^−1^, 45 min). The samples were washed again, resuspended in PBS, and analyzed using a BD LSR Fortessa flow cytometer (BD Biosciences). The results were analyzed with the FlowJo software (FlowJo LLC).

##### Thyroid Cancer Models

All animal experimental procedures and protocols had been approved by the Institutional Animal Care and Use Committees (University of Wisconsin–Madison and Renji Hospital, School of Medicine, Shanghai Jiao Tong University). Since the ATC cell line THJ‐16T has an excellent tumor take rate of 100%,^[^ ^26^ ^]^ we chose this cell line to establish subcutaneous and orthotopic ATC models in nude mice (4–5 weeks; Envigo). Orthotopic ATC models were also established using the newly‐developed ultra‐immunodeficient R2G2 mice (B6;129‐Rag2^tm1Fwa^IL2rg^tm1Rsky^/DwlHsd, 4–5 weeks; Envigo). For subcutaneous ATC models, 5 × 10^6^ THJ‐16T cells (per mouse) were mixed in sterile PBS and matrigel matrix (Corning) with a ratio of 1:1 and injected into the right posterior flank of the experimental mice. Whereas for orthotopic ATC models, 0.5–1 × 10^6^ THJ‐16T or THJ‐16T^Luc^ cells or (per mouse) suspended in sterile PBS were gently injected into the left thyroid bed after surgical exposure of the thyroid.^[^ ^51^ ^]^

##### Fluorescent Imaging and Image‐Guided Surgery

The burden of orthotopic tumors was monitored by fluorescent imaging with IRDye 800CW‐pertuzumab, an NIR probe that was previously described.^[^ ^26^ ^]^ IRDye 800CW‐ALT‐836 was developed in a similar way with a dye‐to‐mAb molar ratio of 10. Serial IRDye 800CW‐ALT‐836 fluorescent imaging (*λ*_ex/em_: 745/800 nm) was carried out using an IVIS Spectrum (Perkin Elmer Inc.), followed by image‐guided resection of the orthotopic ATCs using a handheld Fluobeam (Fluoptics).^[^ ^52^ ^]^ To detect the baseline burden of the orthotopic ATCs, D‐luciferin (3 mg per mouse) was injected into the THJ‐16T^Luc^‐bearing nude mice and bioluminescence imaging was acquired with the IVIS Spectrum 10 min later. The data were analyzed using the Living Image 4.5.5 (IVIS Imaging Systems) software.

##### ^64^Cu‐Labeling and Small Animal PET Imaging

The TF‐specific mAb ALT‐836 and a nonspecific human IgG (Invitrogen) were used to conjugate TF‐targeted probe and isotype control probe, respectively. The antibodies were first linked to 2‐S‐(4‐isothiocyanatobenzyl)‐1,4,7‐triazacyclononane‐1,4,7‐triacetic acid (*p*‐SCN‐Bn‐NOTA; Macrocyclics) as previously described.^[^ ^32^ ^]^ For radiolabeling, 50–100 µg of NOTA‐mAb was reacted with 74–148 MBq of ^64^CuCl_2_ in 300 µL of sodium acetate buffer (0.1 m, pH 4.5) at 37 °C for 1 h under constant shaking (500 rpm). The final ^64^Cu‐NOTA‐mAb was separated from the free activity using equilibrated PD‐10 columns (GE Healthcare Life Sciences) with PBS as the mobile phase.^[^ ^53^ ^]^ On average, 3.7–7.4 MBq of filtered ^64^Cu‐NOTA‐ALT‐836 or ^64^Cu‐NOTA‐IgG was injected to each mouse and PET imaging was acquired with Inveon PET/CT scanner (Siemens). PET data were reconstructed on the Inveon Acquisition Workplace and analyzed using the Inveon Research Workplace.^[^ ^54^ ^]^ MIP and coronal PET images were given to showcase the overall and regional distribution of the radiotracers, respectively. After analyzing the ROI on decay corrected PET images, quantitative data reflecting the dynamic change of the radiotracers in the blood circulation and other major organs were given in terms of percent of injected dose per gram of tissue (% ID per g).

##### ^131^I‐Labeling

To explore the therapeutic effect of TF‐targeted regimens, treatment studies were further designed. Nude mice bearing orthotopic THJ‐16T or THJ‐16T^Luc^ tumors were randomly divided into five groups, including control group (*n* = 7), ^131^I‐ALT‐836 treatment group (*n* = 7), ^131^I‐IgG treatment group (*n* = 5), ALT‐836 treatment group (*n* = 5), and free ^131^I treatment group (*n* = 5). ^131^I‐ALT‐836 and ^131^I‐ALT‐IgG were prepared according to a previously reported protocol.^[^ ^55^ ^]^ Briefly, to a glass vial pre‐coated with 50 µg of IODO‐GEN (Pierce), 50 µL 0.5 m Na_2_HPO_4_, 400 µL antibody solution (400–600 µg of mAb in 0.1 m Na_2_HPO_4_), and 148–185 MBq of ^131^I solution (Shanghai Xinke Pharmaceutical Co. Ltd) were successively added. The mixture was incubated on a constant shaker for 4 min at room temperature, followed by addition of 100 µL of ascorbic acid (25 mg mL^−1^, pH = 5) and incubation for another 5 min. Free ^131^I was removed by PD10 columns with 0.9% NaCl/ascorbic acid (5 mg mL^−1^, pH = 5) as the mobile phase. Radiolabeling efficiency and radiochemical purity of the collected radiopharmaceutical were assessed by iTLC (Biodex Medical System) and HPLC (Agilent).

##### Radioimmunotherapy

Treatment was initiated two weeks after tumor cell inoculation. Five groups were set up to compare the treatment efficacy of ^131^I‐ALT‐836 RIT with that of other counterparts. Mice in the control group were treated with tail vein injection of sterile saline (100 µL per mouse). Mice in the mAb treatment group were treated with a single tail vein injection ALT‐836 (125 µg per mouse). ATC‐bearing mice were given 0.1% potassium iodide‐containing drinking water two days prior to the administration of radioactive regimens. For the two RIT groups, mice were intravenously injected with ^131^I‐ALT‐836 (478 ± 36.17 µCi, *n* = 7) or ^131^I‐IgG (395 ± 84.32 µCi, *n* = 5), respectively. Whereas only free ^131^I (445 ± 31.94 µCi, *n* = 5) was intravenously injected to the mice in the ^131^I treatment group. Toxicity was monitored by measuring body weight, and the endpoint criterion was defined as the presence of severe dyspnea. Treatment efficacy was evaluated by performing fluorescent imaging and constructing survival curves.

##### Biodistribution Studies

After termination of the PET imaging, experimental mice were sacrificed and major tissues (including blood) were collected and weighed. The activity (counts per minute) was counted with a calibrated *γ*‐counter (Perkin Elmer Inc.) and tissue uptake was calculated and given in terms of % ID per g. To examine the in vivo circulation, stability, and tumor‐targeting efficacy of ^131^I‐ALT‐836 and ^131^I‐IgG, a lower dose of the agents (156.13 ± 28.68 for ^131^I‐ALT‐836 and 219.63 ± 42.29 for ^131^I‐IgG, *n* = 4 for each group) was injected and biodistribution studies were performed two days post‐injection.

##### Histopathology Studies

To detect TF expression and vasculature, tumor tissues were fixed in 10% paraformaldehyde and sliced tumor sections (10 µm) were stained with 20 µg mL^−1^ of ALT‐836 and 10 µg mL^−1^ of CD31/PECAM‐1 antibody (MEC13.3; Novus), respectively. The washed sections were then stained with 5 µg mL^−1^ of Alexa Fluor 488‐labeled goat anti‐human IgG (Invitrogen) and 5 µg mL^−1^ of Cy3‐labeled donkey anti‐rat IgG (Jackson ImmunoResearch Laboratories, Inc.). After washing, the sections were mounted with the UltraCruz Hard‐set Mounting Medium containing 1.5 µg mL^−1^ of DAPI (Santa Cruz Biotechnology). All the fluorescent images were acquired using a Nikon A1R confocal microscope.^[^ ^54^ ^]^ To detect the involvement of the adjacent structures, orthotopic tumors together with thyroid, trachea, muscle, and larynx were carefully resected at necropsy and used for H&E staining as previously described.^[^ ^51^ ^]^

##### Statistical Analysis

Statistical analyses were performed using the GraphPad software. All data are presented as the mean value ± SD or value ± SEM. Group data were compared using the two‐tailed Student's *t*‐test, and multiple comparisons of grouped data were calculated using two‐way ANOVA. Survival was calculated using Kaplan–Meier curves. *p* < 0.05 was considered statistically significant.

## Conflict of Interest

The authors declare no conflict of interest.

## Supporting information

Supporting Information
