# Supplementary material for: Tissue Factor‐Targeted ImmunoPET Imaging and Radioimmunotherapy of Anaplastic Thyroid Cancer
Source: Adv Sci (Weinh). 2020 May 17;7(13):1903595. doi: 10.1002/advs.201903595 (PMC7341097; doi:10.1002/advs.201903595)
Supplement: Supplementary file 1 — Supporting Information [file ADVS-7-1903595-s001.pdf]

Copyright WILEY-VCH Verlag GmbH & Co. KGaA, 69469 Weinheim, Germany, 2020.

**Supporting Information**

**Tissue Factor-targeted ImmunoPET Imaging and Radioimmunotherapy of Anaplastic Thyroid Cancer**

Weijun Wei[1,2,#], Qiufang Liu[3,#], Dawei Jiang[2], Haitao Zhao[1], Christopher J. Kutyreff[2], Jonathan W. Engle[2], Jianjun Liu[1,*], Weibo Cai[2,4*]

[1] Department of Nuclear Medicine, Renji Hospital, School of Medicine, Shanghai Jiao Tong University, 1630 Dongfang Rd, Shanghai 200127, China

[2] Departments of Radiology and Medical Physics, University of Wisconsin – Madison, Madison, Wisconsin 53705, United States

[3] Department of Nuclear Medicine, Fudan University Shanghai Cancer Center, Fudan University, 270 Dongan Rd, Shanghai 200032, China

[4] University of Wisconsin Carbone Cancer Center, Madison, Wisconsin 53705, United States

[#]: The authors contributed equally to this work

[*]**:** Correspondence authors:

**Prof. Jianjun Liu**

**Email:** nuclearj@163.com

**Tel:** +86-021-68383963

**Address:** Department of Nuclear Medicine, Renji Hospital, School of Medicine, Shanghai Jiao Tong University, 1630 Dongfang Rd, Shanghai 200127, China

**Prof. Weibo Cai**

**E-mail:** wcai@uwhealth.org

**Tel:** 608-262-1749

**Address:** Room 7137, 1111 Highland Avenue, University of Wisconsin – Madison, Madison, WI 53705-2275, United States

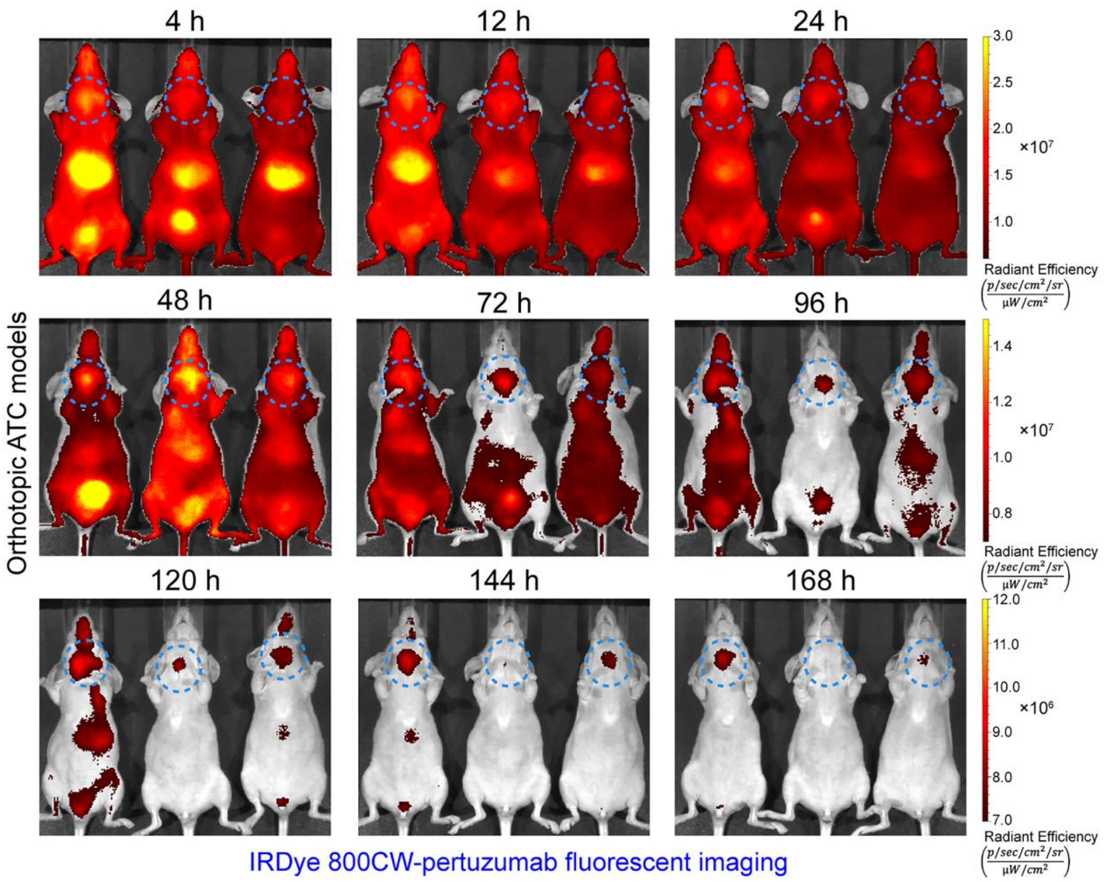


**Supplemental Figure 1**. IRDye 800CW-pertuzumab fluorescent imaging of orthotopic anaplastic thyroid cancer (ATC) models two weeks after tumor cell inoculation. The thyroid areas were indicated by blue dashed circles. The results showed an obvious fluorescent signal in the thyroid areas, indicating the growth of the injected THJ-16T cells. Nude mice were used to establish the orthotopic ATC models in this group.

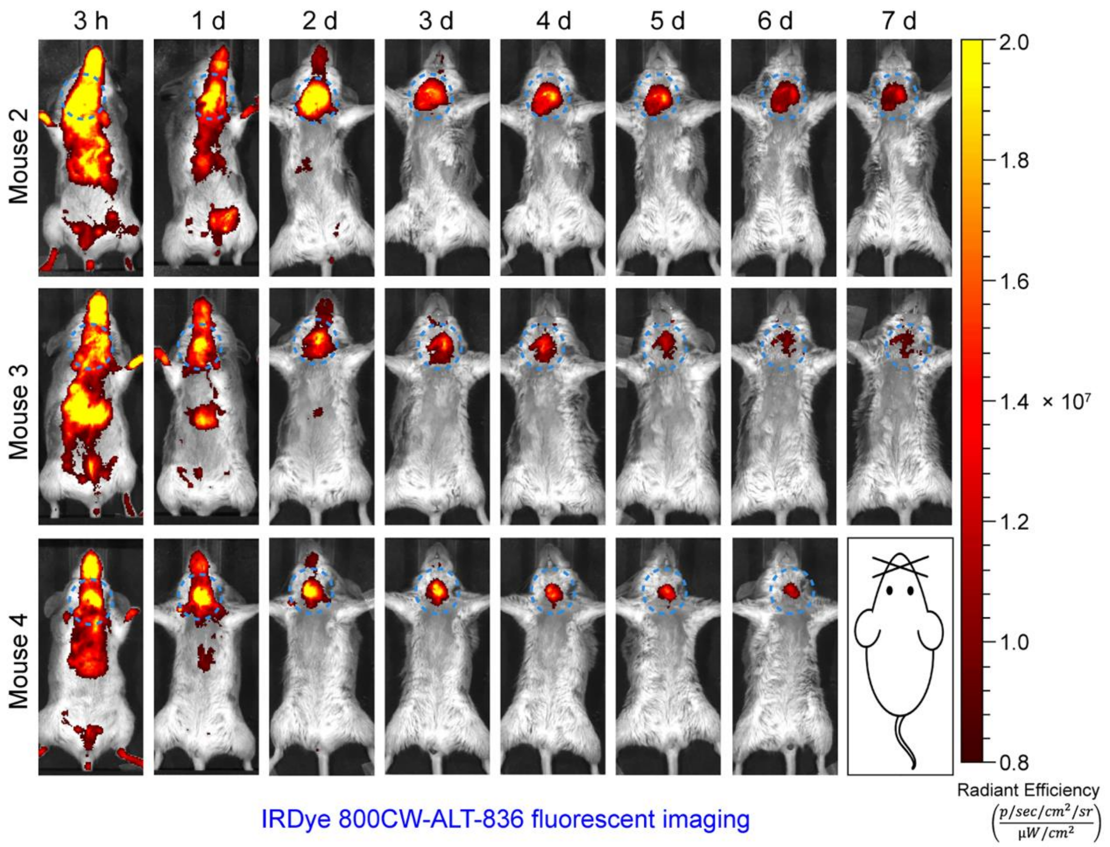


**Supplemental Figure 2**. IRDye 800CW-ALT-836 fluorescent imaging of orthotopic anaplastic thyroid cancer (ATC) models four weeks after tumor cell inoculation. It is notable that R2G2 mice (Envigo) were used in this group to establish orthotopic ATC models. Image-guided surgeries were carried out following fluorescent imaging at the terminal time-point, that is, seven days after injection of IRDye 800CW-ALT-836. The tumors were outlined by blue dashed circles.

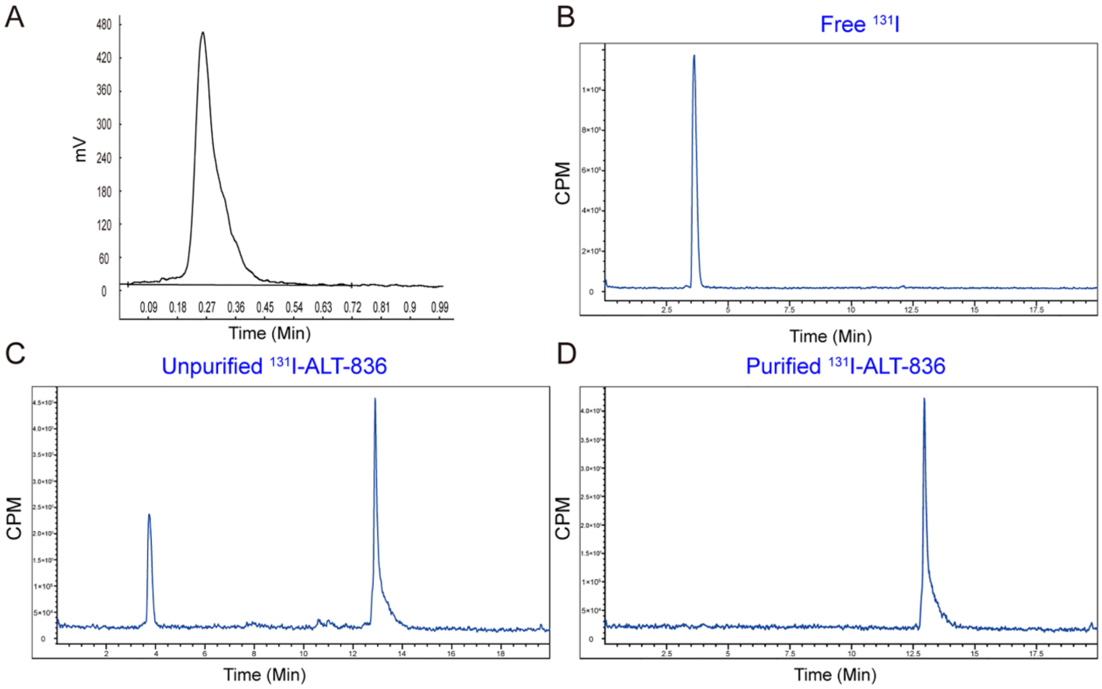


**Supplemental Figure 3**. Characterization of $^{131}$I-ALT-836. (A) iTLC showed an excellent radiochemical purity of 100%. HPLC analysis of (B) free $^{131}$I, (C) unpurified $^{131}$I-ALT-836, and (D) purified $^{131}$I-ALT-836. After purification with the PD10 desalting column, HPLC analysis yielded a single peak with a retention time of 13 min, confirming the purity of the $^{131}$I-ALT-836.

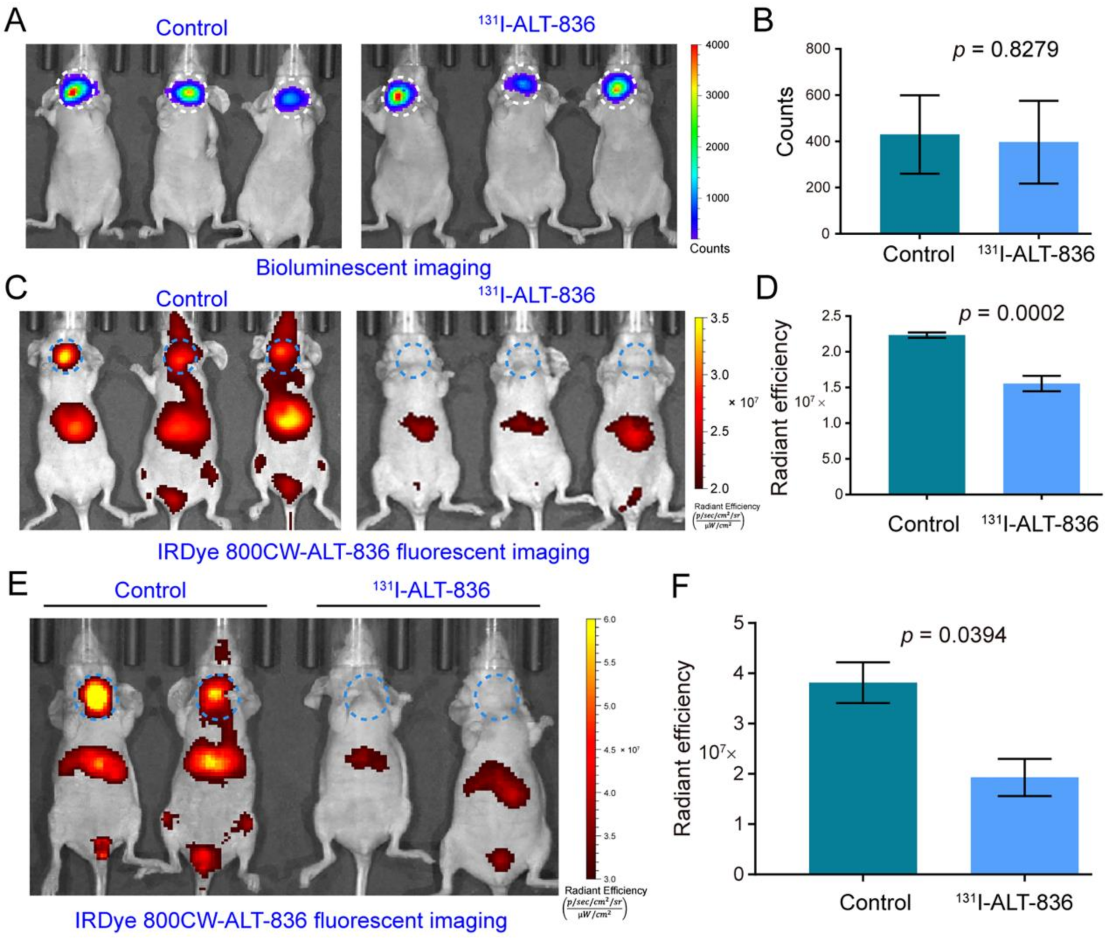


**Supplemental Figure 4**. (A) Bioluminescence imaging of mice in the control and $^{131}$I-ALT-836 treatment groups before initiation of treatment. Tumors were outlined by white dashed circles. (B) Quantitative analysis showed comparable signals in these two groups. (C) Fluorescent imaging with IRDye 800CW-ALT-836 26 days after the treatment. Tumors and thyroid areas were indicated by blue dashed circles. (D) Quantitative analysis of fluorescent intensity in the control and $^{131}$I-ALT-836 treatment group. (E) Fluorescent imaging with IRDye 800CW-ALT-836 41 days after the treatment. Tumors and thyroid areas were indicated by blue dashed circles. (F) Quantitative analysis of fluorescent intensity in the control and $^{131}$I-ALT-836 treatment group.

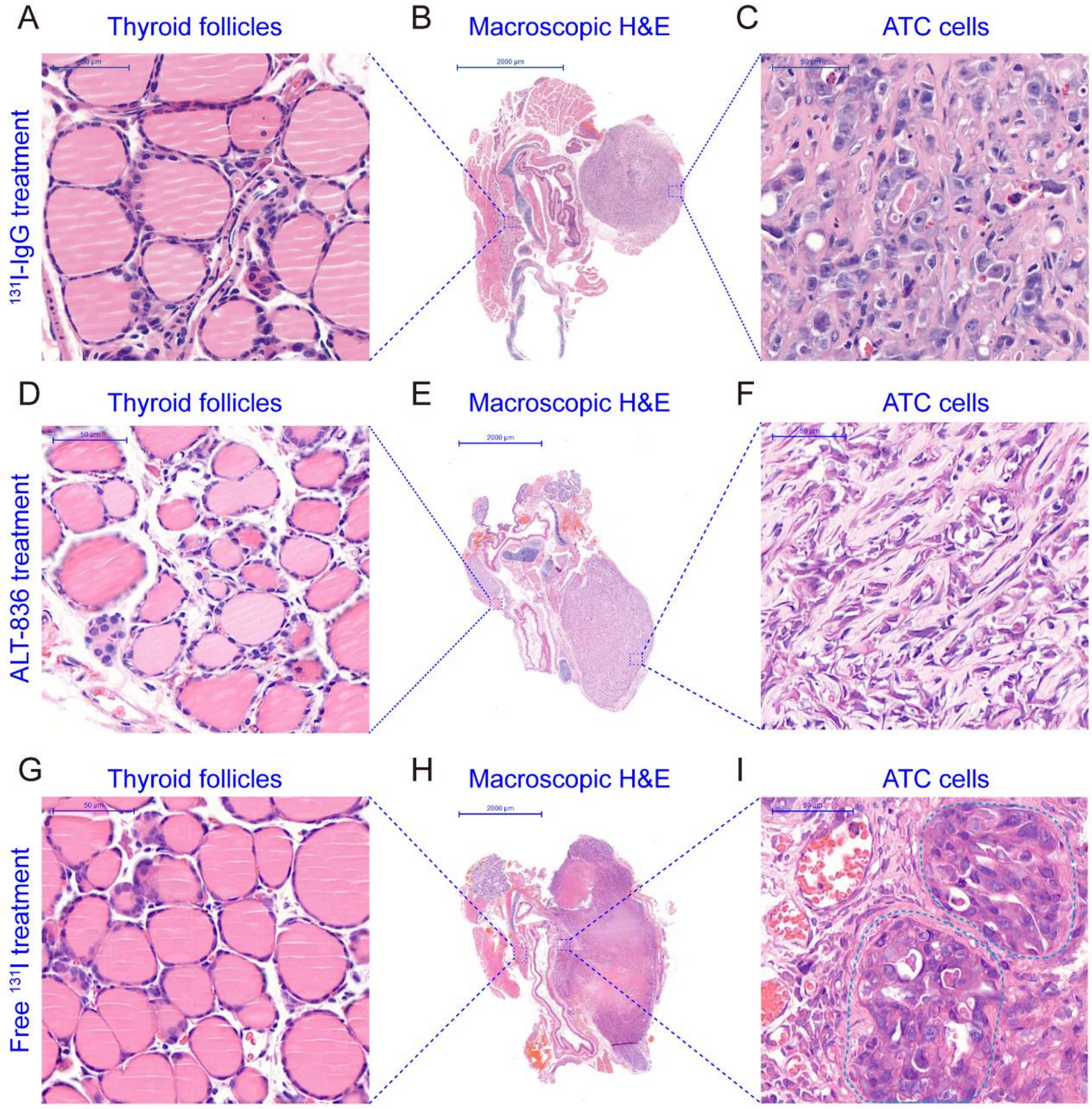


**Supplemental Figure 5**. H&E staining of tumor tissues in the $^{131}$I-IgG treatment group, ALT-836 treatment group, and free $^{131}$I treatment group. While right thyroids in the given cases (A, D, and G) remained intact and typical thyroid follicles were filled with colloid, left thyroid areas (C, F, and I) in these cases were heavily effaced by the THJ-16T cells, as exemplified by ***Supplemental Figure 5I*** where two thyroid follicles were infiltrated and filled by THJ-16T cells (dark green dashed circles).

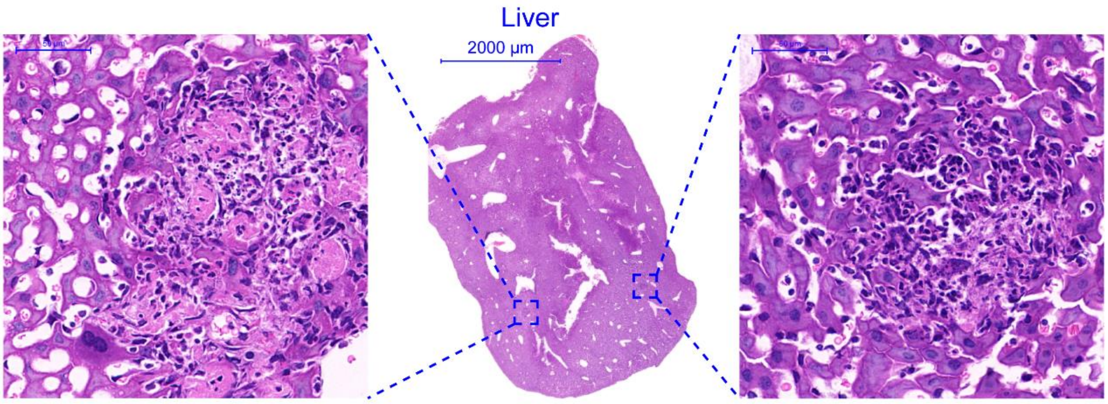


**Supplemental Figure 6**. H&E staining of liver tissue in the $^{131}$I-ALT-836 treatment group. A small proportion of liver cells underwent degradation and necrosis following the therapy.

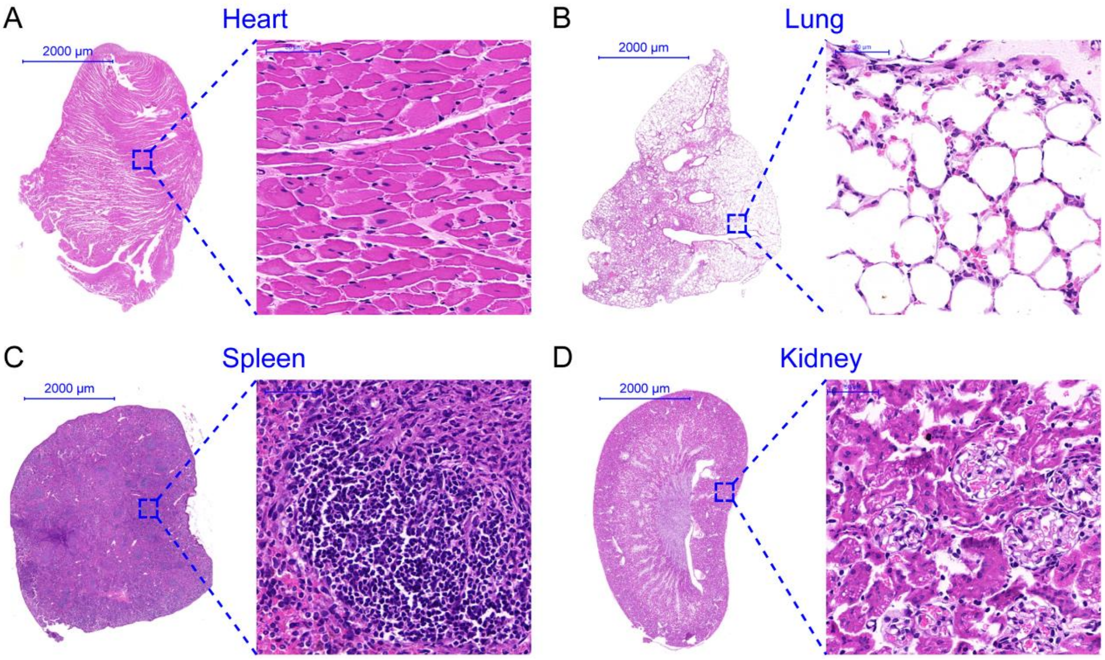


**Supplemental Figure 7**. H&E staining of heart, lung, spleen, and kidney in the $^{131}$I-ALT-836 treatment group. No obvious damage or toxicity was observed in these organs.

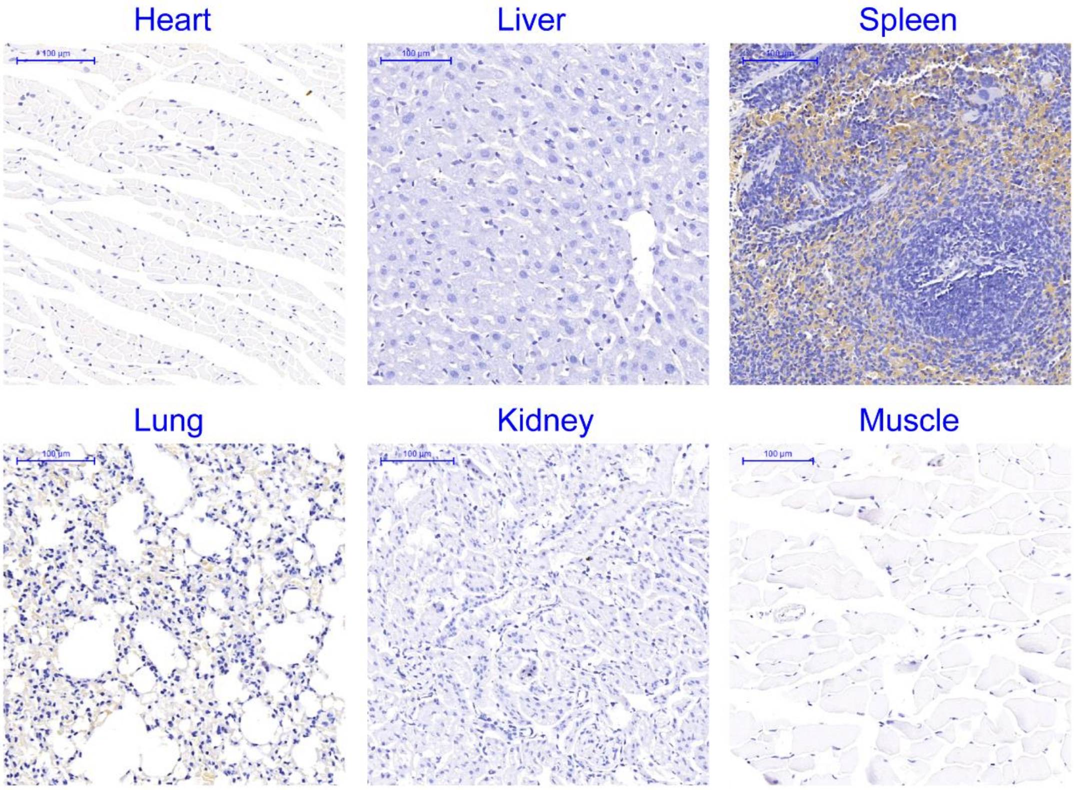


**Supplemental Figure 8**. Immunohistochemistry staining of normal murine organs or tissues showed negligible expression of tissue factor. Positive staining in the spleen and lung was largely caused by the postive staining of blood cells.

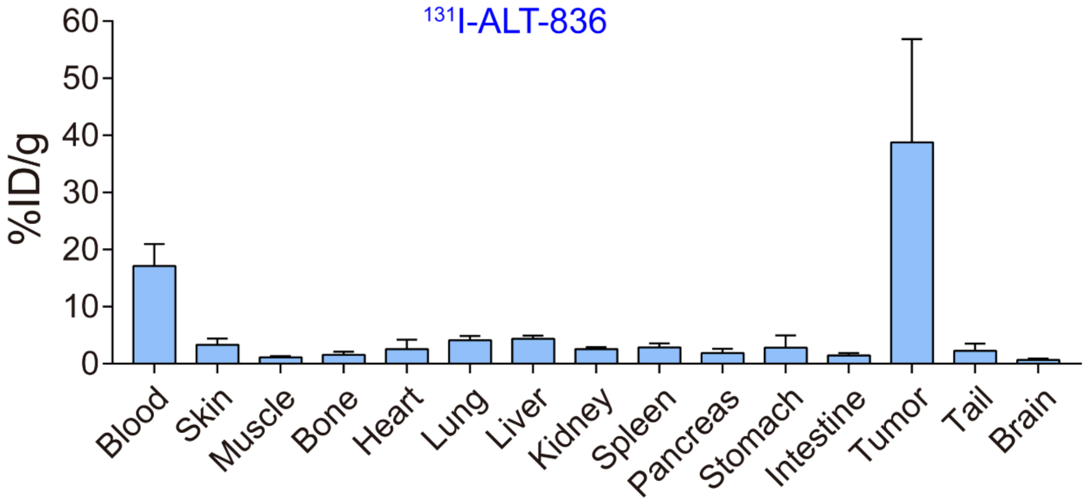


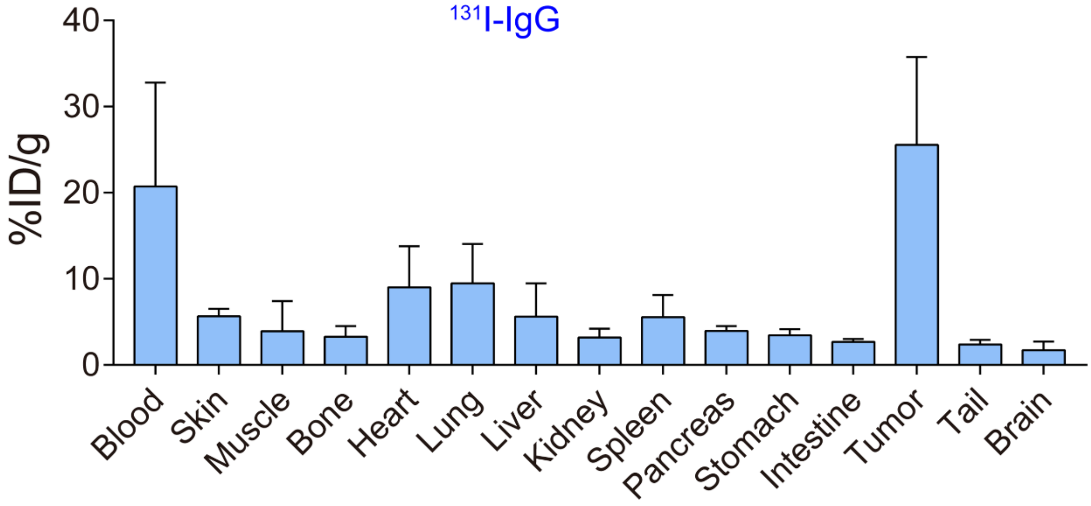


**Supplemental Figure 9**. Biodistribution data showing the overall distribution of $^{131}$I-ALT-836 and $^{131}$I-IgG in the THJ-16T-bearing mice (n = 4 for each group).
